# A Long-Term National Survey of Compliance with the Ban on Selling Tobacco Products to Adolescents of Taiwan’s Tobacco Hazards Prevention Act

**DOI:** 10.3390/healthcare10102077

**Published:** 2022-10-19

**Authors:** Shan-Ru Li, Yu-Lung Chiu, Ching-Huang Lai, Ya-Mei Tzeng, Yi-Yin Lai, Wan-Lu Liao, Kuei-Chia Chang, Rong-Dar Wu, Li-Fen Lei, I-Teng Huang, Senyeong Kao

**Affiliations:** 1 Graduate Institute of Life Sciences, National Defense Medical Center, Taipei City 114, Taiwan; 2 School of Public Health, National Defense Medical Center, Taipei City 114, Taiwan; 3 Army 257th Infantry Brigade, Chiayi County, Chiayi City 622, Taiwan; 4 Consumers’ Foundation Chinese Taipei, Taipei City 106, Taiwan; 5 Department of Agricultural Economics, National Taiwan University, Taipei City 106, Taiwan

**Keywords:** adolescent, compliance, mystery customers, retailer, tobacco hazards prevention act

## Abstract

The data on long-term trends and factors of tobacco retailers’ compliance in Taiwan are limited. The new regulations of the Tobacco Hazards Prevention Act were established in 2009. Now, the government is planning to raise the minimum legal age (MLA) for purchasing tobacco products from 18 to 20, so the results of this study will be an important reference to promote new regulations in the future. We carried out an observational mystery shopping study design and data were collected from 2009 to 2019. In total, 6320 undercover tests were conducted to investigate selling by tobacco retailers to persons aged less than 18 years by an impartial third party annually. Logistic regression was used to analyze the factors influencing compliance by adjusting test variables and independent variables. The compliance rate increased by 8.4% annually and was better among tests conducted during summer vacation (AOR = 1.324), chain convenience stores (AOR = 3.651), supermarkets or hypermarkets (AOR = 1.973), and verifications with age (AOR = 15.345). It is the first study to explore long-term and national tobacco retailers’ enforcement effects by an impartial third party in Asia. The findings suggest that local health agencies should enhance enforcement on those stores which were tested during non-summer holidays and weekends, betel nut stands, and grocery stores.

## 1. Introduction

Smoking is one of the significant causes of death [1,2,3]. However, smoking affects adolescent health slowly, and the morbidity rate for adolescents is lower than that for adults, leading adolescents to overlook the effects of smoking on their health. In addition, smokers are prone to respiratory disease symptoms, thus consuming many medical resources [4]. Adolescents exposed to tobacco products have a higher risk of becoming smokers and may even become users of multiple tobacco products (including novel and emerging tobacco products). These problems have received considerable research attention in various countries [5,6,7].

Various tobacco control policies have been stipulated in the Framework Convention on Tobacco Control (FCTC) passed by the World Health Organization (WHO) in 2003. Among them, laws have a more substantial binding effect [8]. Although Taiwan is not a contracting party to the WHO, considerable resources are invested in Taiwan for tobacco hazards prevention following such international prevention measures. In addition, several related smoking regulations stipulate that adolescents are not allowed to smoke and that tobacco products should not be sold to adolescents. However, reports have revealed smoking among adolescents; regarding the sources of tobacco products, the majority of junior high school students and senior high school/vocational high school students purchased tobacco products by themselves (45.7% and 71.3%, respectively). The majority of adolescent students purchased tobacco products from grocery and traditional stores (55.8%) and convenience stores (54.3%) [9]. Clearly, tobacco retailers were not compliant with the relevant laws and regulations; therefore, students could access tobacco products by themselves. Many countries have increased the minimum legal age (MLA) for accessing tobacco products to 21, and the health authority of Taiwan is also planning to raise the legal age for tobacco purchase to 20. Therefore, it’s important to know the effect of enforcement and factors influencing retailers’ compliance to enact strategies to prevent tobacco retailers from illegally selling to minors.

A literature review revealed that the influencing factors of compliance included holidays, types of stores, age verification, and clerks’ characteristics [10,11,12,13,14]. However, few studies have evaluated the potential effects of local characteristics together with tobacco access and usage among adolescents, including adolescents’ smoking rate, exposure rate to secondhand smoke at home, and juvenile offender rate [15]. In addition, the insufficient data of national research about the long-term effects of enforcement on tobacco retailers means that the effects of the tobacco hazards prevention policies on counties, cities, and different types of stores are less well understood.

### Research Questions (RQ)

It has been a decade since the implementation of the new Tobacco Hazards Prevention Act, which has been in force from 2009 to the present. We aimed to explore the long-term trend from 2009 to 2019 and the overall factors influencing enforcement effects in this ten-year period on selected tobacco retailers in Taiwan.

## 2. Materials and Methods

The tests were conducted annually by a credible third party commissioned by the health promotion authority in Taiwan to understand the effectiveness and problems of local governments in promoting tobacco prevention laws, to establish a database as a reference in next year’s tobacco prevention budget, and for future improvement and policy planning in implementing tobacco prevention laws. This impartial third party has branches in the North, Central, and South regions of Taiwan, and it is sufficiently experienced and well-resourced to implement the tests. With this budget, a total of 6320 tests were conducted from 2009 to 2019, with 225 tests in 2009, 375 tests in 2010, 550 tests in 2011–2012, and 660 tests in 2013–2019. Cross-sectional data were collected every year, and retailers were tested once in one year. Our tests were similar to a previous study that analyzed changes in retailers’ compliance in Hawaii from 1996–2013 [16], but the total sample sizes of the previous studies [16,17,18,19] were smaller than ours.

This study adopted an observational mystery shopping study design to investigate the selling of tobacco products to persons aged less than 18 years in various tobacco-selling sites through disguise tests. (Table S1). This study examined four main types of tobacco-selling sites in Taiwan: convenience stores of the four leading chains, betel nut stands, supermarkets or hypermarkets, and grocery stores.

The sample size was calculated by the formula of Kish (1965), which was calculated by the confidence level (97%), the overall legal compliance rate of stores in the previous year (P), and the sample deviation (D); and the minimum sample size was 30 for each county and city [20]. Then, due to administrative procedures and to facilitate comparison, the sample size in the disguise test for each county/city each year was fixed at 30. The test task was divided into three stages: the period from April to May, from June to July, and from August to September. Random sampling was used in each county and city to select 10 tobacco-selling sites at each stage. For these three stages, tobacco-selling locations were classified into the following types: seven convenience stores of the four leading chains, seven betel nut stands, five supermarkets or hypermarkets, and eleven public places selling tobacco products, totaling 30 places.

### 2.1. Process of Disguise Tests

The disguise tests were employed to evaluate the selling of tobacco products to persons aged less than 18 years in the four main types of tobacco-selling places. Only students over the age of 18 were recruited as testers. Testers received standardized training on camouflage testing, such as the recording process, standardized conversations, and responding to emergencies.

Before the formal test, a pretest, including the test procedure, standard dialogue, and recording process, was held to help testers become familiar with the survey process. Then, two testers 18 years or older formed a group to survey the actual test. For example, one tester who wore an old high school uniform entered a convenience store to buy cigarettes and made a recording. The other person was responsible for taking photos outside the store and filling out a survey sheet covering the store name and address and detailing whether age was inquired about or whether identity document presentation was requested in the store.

### 2.2. Dependent Variables

The test results were divided into a noncompliance group and a compliance group. The noncompliance group included: “clerks selling tobacco products without verifying customers’ age” and “clerks inquired customer’s age and customers replied they did not bring identity documents, but clerks still selling tobacco products to them”.

The compliance group included: “clerks inquired customers’ age and refusing to sell tobacco products to those who replied they did not bring identity documents” and “clerks directly refused to sell tobacco products to customers”.

### 2.3. Test Variables

Test variables were collected from the tests and included summer vacation grouping, weekdays/weekends grouping, types of stores, and age verification.

In terms of summer vacation grouping, according to the Ministry of Education’s classification of school years, semesters, and holidays for students at all school levels, April through June and September through October were merged into the non-summer vacation period. In contrast, July through August was integrated into the summer vacation period.

Regarding weekdays/weekends grouping, the test dates initially recorded in the database were compared with the work calendar released by the Directorate-General of Personnel Administration; Monday through Friday were merged into weekdays, and Saturday through Sunday were integrated into weekends [10].

Types of stores included four types: chain convenience stores, betel nut stands, supermarkets and hypermarkets, and grocery stores.

The response of selling tobacco products without checking the age of customers was considered as no age verification. Those responses having inquired customer’s age by ID were considered as having age verification.

### 2.4. Independent Variables

Independent variables represented the local characteristics, including the proportion of divorce rate (‰), exposure rate to secondhand smoke at home (%), juvenile offender rate (criminals per 100,000 persons), the smoking rate among adolescents (senior and vocational high school, and junior high school students) (%), male adolescents (%), and county/city grouping [21,22,23,24,25,26]. The data of the first five variables were based on publicly accessible statistics from 22 counties and cities from 2009 to 2019 that were provided on governmental web pages.

Regarding the variable of county/city, the 22 counties and cities were divided into four groups by quartile calculation and based on the average disposable income of the county/city; the group was made based on the average disposable income per household (NTD) in the category of household income and expenditure, according to the Directorate General of Budget, Accounting, and Statistics of Executive Yuan. Those with the highest disposable income were ranked the top 25% of the year; those with the second highest disposable income were ranked the top 26–50% of the year; those with the second lowest disposable income were ranked the top 51–75% of the year; those with the lowest disposable income were ranked the top 76%–100% of the year [21].

### 2.5. Statistical Analysis

Microsoft Excel 2016 and SPSS 22.0 were used for data compilation and analysis. Noncompliance and compliance were considered the dependent variables. Year, county/city, summer vacation grouping, weekdays/weekends, types of stores, and age verification were treated as the test variables. Proportion of male adolescents, the smoking rate among adolescents, divorce rate, exposure rate to secondhand smoke at home, and juvenile offender rate were the independent variables. Curve estimation was used to analyze the time trend of compliance rate from 2009 to 2019. Chi-squared test and independent sample t-test were used to analyze the differences of test variables, independent variables and dependent variables. If the variable was significant in the difference analysis, it was adjusted in the final regression analysis. Finally, we used logistic regression analysis to examine the influencing factors of dichotomous dependent variables, with adjustment for the test variables and independent variables (except juvenile offender rate). We also showed the Nagelkerke’s R squares for goodness-of-fit results, β and standard errors, Wald statistic, odds ratio (OR), and 95% confidence interval. A *p*-value < 0.05 was considered statistically significant.

## 3. Results

A total of 6320 tests were used in this study, and the compliance rate was 53.9%. Table 1 shows the compliance and noncompliance of independent variables and test variables. In the independent variables, exposure rate to secondhand smoke at home (24.0 ± 4.69 vs. 23.5 ± 4.30), smoking rate among senior and vocational high school (10.6 ± 3.86 vs. 9.8 ± 3.99) and junior high school (5.5 ± 3.24 vs. 5.1 ± 3.05) students were significantly higher in the noncompliance group. In the test variables, the highest compliance rate was observed in 2017 (68.0%); the counties/cities with the average second highest disposable income (56.1%) were those with the highest compliance rate; tests during summer vacation (57.7%) were better than during the non-summer vacation period (51.6%); tests on weekdays (58.0%) were better than weekends (47.5%); chain convenience stores (68.7%) had a higher compliance rate than others; and sellers that verified age had a higher compliance rate (88.9%) than non-verifying ones.

**Figure 1:**
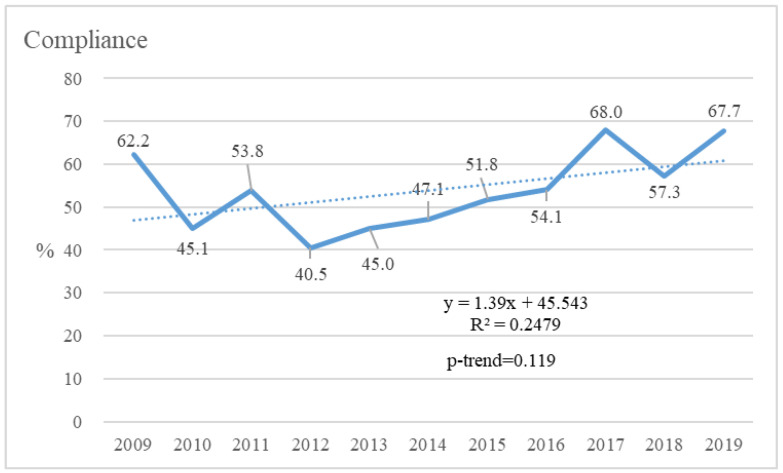
The trend of compliance rate from 2009 to 2019.

The compliance rate in 2009 was 62.2% but decreased to 45.1% in 2010, and the lowest compliance rate was in 2012 (40.5%). The annual trend of the overall compliance rate was from 62.2% in 2009 to 67.7% in 2019 (*p* for trend = 0.119) (Figure 1).

Since juvenile offender rate had no difference with the test result, the remaining variables were further analyzed in the logistic regression model. Among the independent variables, higher exposure rate to secondhand smoke at home (Adjusted odds ratio = 0.977, 95% CI = 0.963–0.991), higher smoking rate among senior and vocational high school students (AOR = 0.964, 95% CI = 0.945–0.984), and male adolescent rate (AOR = 0.887, 95% CI = 0.818–0.962) were associated with lower compliance.

Among the test variables, the years of survey (AOR = 1.085, 95% CI = 1.051–1.120), inspection during summer vacation (AOR = 1.324, 95% CI = 1.163–1.507), chain convenience stores (AOR = 3.651, 95% CI = 3.099–4.301), supermarkets or hypermarkets (AOR = 1.973, 95% CI = 1.625–2.396), and sellers verified with age (AOR = 15.345, 95% CI = 13.159–17.893) were associated with better compliance; however, the county/city with the average highest disposable income (AOR = 0.572, 95% CI = 0.468–0.698) and second highest disposable income (AOR = 0.818, 95% CI = 0.673–0.993), and tests on weekends (AOR = 0.784, 95% CI = 0.688–0.894) were associated with worse compliance (Table 2).

## 4. Discussion

The findings showed that a higher compliance rate was associated with the year of test, second highest disposable income, test during summer vacation and weekdays, chain convenience stores, supermarkets or hypermarkets, and the group with age verification. From 2009 to 2019, the overall compliance rate was 53.9% and decreased by year; the trend was the same as the Sweden study [17]. Additionally, the compliance rate in 2009 (62.2%) was higher than the Switzerland (17.8%) and Hong Kong studies (27.0%) [18,27], but much lower than the United States studies (73.4–95.7%) [10,11,16]. Moreover, we found the compliance rate was lower than FDA inspection results in recent years [28]. This may be due to the fact that this study was not conducted by law enforcement officers but by two pseudo-underage students who were older than 18 years.

In terms of the test year, the data indicated that the compliance rate was lower in 2010 and 2012 to 2015; the same trend has been reported in the Washington study about implementing new tobacco hazards prevention policies from 2001 to 2005 [11]. On the other hand, the noncompliance rate was significantly lower between 2017 and 2019, probably due to the increase in the tobacco tax in June 2017 in Taiwan. Basing on a smoking behavior theoretical model, raising the tobacco tax or price of cigarettes could stop adolescents’ smoking habit. In other words, raising the tobacco tax was effective at decreasing the smoking rate among adolescents [15,29]. Additionally, raising the minimum legal purchase age could decrease the sales of retailers [28].

In our study, higher compliance rate was observed in tests during summer vacation than non-summer vacation. Research has revealed that adolescent smoking mostly occurs during this time [30], and according to the National Police Agency, Ministry of the Interior, the number of juvenile suspects was higher during summer vacation. Thus, the authority has implemented the Protect Adolescents in Summer project by combining local and nongovernmental resources to reduce juvenile delinquency. The project also involved enhanced joint inter-ministerial inspection to prevent drug abuse and advocate for tobacco hazard prevention [31], and thus improve retailers’ alertness and compliance during summer vacation.

Similarly, the study results revealed that tests on weekdays had a higher probability of compliance with the regulation. According to research, it is probably because clerks had less education training and strict supervision on weekends; in addition, clerks who worked on weekends were relatively younger, so they were more likely to sell tobacco products to a person the same age as their peers [10].

Chain convenience stores and supermarkets or hypermarkets were more likely to be compliant with the regulation than grocery stores. Silver et al. (2016) presumed that regulations of supervision and training in chain stores would be stricter than other stores [12], whereas studies in the United States have revealed lower compliance in convenience stores selling gas or stores attached to gas stations than chain convenience stores [10,11,14], because they were mostly privately- or family-run, and they mostly did not implement strictly laws and regulations [14]. According to a European study exploring the effectiveness of tobacco hazards prevention policies among adolescents, interviewed students stated that, due to the stricter supervision mechanism in chain convenience stores, supermarkets, and hypermarkets (for example, a clerk could be punished or expelled for selling tobacco products to adolescents); adolescents tended to access tobacco products through other approaches, such as small and non-chain tobacco retailers including local stores, stores attached to gas stations, and news-stands [13]. In addition, the betel nut stand is a unique style of store in Taiwan. The results revealed that betel nut stands’ compliance rate was the worst, indicating that relevant competent authorities should further enhance health education and inspection for betel nut stands.

In this study, clerks checking the age of purchasers was an influencing factor of compliance, as in other studies [10,11]. During the tests, when clerks asked the testers’ age, they had to reply, as a standardized procedure, that they did not bring identity documents or they were purchasing for their family or friends. However, adolescents are likely to lie about their age when purchasing tobacco products. According to a prior study, school uniforms are highly recognizable and easily lead to clerks’ alertness. Thus, compliance rate was higher when undercover testers wore school uniforms rather than casual outfits [27]; the rate of age verification was 35.8%. In other countries, the rate ranged from 12.6% to 83.3% [11,12,16], and the rate of asking to show identity documents was even more than 90% [31]. In our tests, the percentage of age verification was lower, probably because the testers were dressed in local school uniforms, but in most studies, testers dressed in casual outfits; thus, the clerks had to further verify the age, which also made the enforcement compliance better.

It was suggested that the competent authorities could prevent retailers from selling tobacco products to adolescents in the future by enhancing regular inspections of stores with lower compliance, conducting amendments, and enhancing education training for staff as well as internal norms in tobacco-selling places [32,33,34].

This study has three limitations. First, testers wore local senior high school uniforms, which avoided deviations in differences in outfit style; however, adolescents who purchase tobacco products generally wear casual outfits. Thus, the compliance was probably overestimated in the study. Second, the data were collected every year, but we did not explore the compliance of repeat tests among some retailers. Thus, it is unknown whether the repeat tests would affect compliance due to heightening alertness or other reasons. Third, the testers were mostly male; therefore, the effects of different sexes on compliance are still unknown.

We suggest future studies could present the location of retailers by using geographic information systems (GIS) and show the compliance results by the degree of urbanization and the distance between stores and schools. The relationship between clerks’ age and compliance should also be further examined. Moreover, tobacco products have become more diverse following the global emergence of new nicotine and tobacco products in recent years, such as heated tobacco products, flavored tobacco, and e-cigarettes. This means the choices of tobacco products for adolescents are no longer limited to cigarettes. Future studies should include the time of survey (such as morning, afternoon, or evening) and the popularity of test sites [10,11,19], particularly internet cafes and other consumer places.

## 5. Conclusions

The sales of tobacco products to adolescents by retailers in Taiwan showed a decreasing trend, indicating that the results were achieved through law enforcement by the central and local health authorities. The influencing factors on compliance were the year of test, second highest disposable income, test during summer vacation and weekdays, chain convenience stores, supermarkets or hypermarkets, and age verification. Therefore, we have some suggestions for the authorities. First, the random joint inspections by central and local health authorities could be conducted during the non-summer vacation to lower retailers’ alertness. Second, owners or supervisors may enhance the education trainings and internal assessments for clerks who work on weekends. Third, health authorities should build a supervision mechanism focused on betel nut stands and grocery stores.

Many countries have increased the minimum legal age for purchasing tobacco products to 21 years, and Taiwan’s government are planning to raise it to 20. Thus, the results of the study may be a reference for the new act, and the authorities could plan and implement new tobacco prevention strategies.

## Figures and Tables

**Table 1:** Association between independent variables and test variables and test results.

Variable	Noncompliance	Compliance	χ^2^/t	*p*
Independent variables				
Divorce rate	2.2 ± 0.40	2.3 ± 0.33	−4.59	<0.001
Exposure rate to secondhand smoke at home	24.0 ± 4.69	23.5 ± 4.30	4.99	<0.001
Juvenile offender rate	755.7 ± 316.44	759 ± 301.09	−0.43	0.671
Smoking rate among senior and vocational high school students	10.6 ± 3.86	9.8 ± 3.99	7.70	<0.001
Smoking rate among junior high school students	5.5 ± 3.24	5.1 ± 3.05	4.72	<0.001
Male adolescent rate	52.4 ± 0.90	52.3 ± 0.70	4.62	<0.001
County/city			16.78	0.001
Highest disposable income	712 (50.7)	693 (49.3)		
Second highest disposable income	650 (43.9)	832 (56.1)		
Second lowest disposable income	793 (45.8)	939 (54.2)		
Lowest disposable income	756 (44.4)	945 (55.6)		
Test variables				
Year			198.79	<0.001
2009	85 (37.8)	140 (62.2)		
2010	206 (54.9)	169 (45.1)		
2011	254 (46.2)	296 (53.8)		
2012	327 (59.5)	223 (40.5)		
2013	363 (55.0)	297 (45.0)		
2014	349 (52.9)	311 (47.1)		
2015	318 (48.2)	342 (51.8)		
2016	303 (45.9)	357 (54.1)		
2017	211 (32.0)	449 (68.0)		
2018	282 (42.7)	378 (57.3)		
2019	213 (32.3)	447 (67.7)		
Summer vacation grouping			22.38	<0.001
Nonsummer vacation period	1892 (48.4)	2018 (51.6)		
Summer vacation	1019 (42.3)	1391 (57.7)		
Weekdays/Weekends			66.27	<0.001
Weekdays	1623 (42.0)	2242 (58.0)		
Weekends	1288 (52.5)	1167 (47.5)		
Types of stores			417.92	<0.001
Chain convenience stores	625 (31.3)	1372 (68.7)		
Betel nut stands	904 (62.0)	554 (38.0)		
Supermarkets, hypermarkets	363 (36.3)	637 (63.7)		
Grocery stores	1019 (54.6)	846 (45.4)		
Age verification			1732.38	<0.001
No	2659 (65.6)	1397 (34.4)		
Yes	252 (11.1)	2012 (88.9)		

**Table 2:** Influencing factors of test results (compliance vs. noncompliance).

Variable	ß (SE)	Wald	Adjusted OR (95% CI)
Independent variables			
Divorce rate	0.112 (0.090)	1.560	1.119 (0.938–1.334)
Exposure rate to secondhand smoke at home	−0.023 (0.007)	10.318	0.977 (0.963–0.991) **
Smoking rate among senior and vocational high school students	−0.037 (0.010)	12.494	0.964 (0.945–0.984) ***
Smoking rate among junior high school students	−0.023 (0.014)	2.701	0.977 (0.950–1.005)
Male adolescent rate	−11.962 (4.120)	8.430	<0.001 (<0.001–0.021) **
County/city			
Highest disposable income	−0.559 (0.102)	30.037	0.572 (0.468–0.698) ***
Second highest disposable income	−0.201 (0.099)	4.130	0.818 (0.673–0.993) *
Second lowest disposable income	−0.088 (0.090)	4.130	0.916 (0.768–1.092)
Test variables			
Year	0.082 (0.016)	25.366	1.085 (1.051–1.120) ***
Nonsummer/Summer vacation			
Summer vacation	0.281 (0.066)	18.097	1.324 (1.163–1.507) ***
Weekdays/Weekends			
Weekends	−0.243 (0.067)	13.118	0.784 (0.688–0.894) ***
Types of stores			
Chain convenience stores	1.295 (0.084)	240.100	3.651 (3.099–4.301) ***
Betel nut stands	−0.161 (0.087)	3.444	0.852 (0.719–1.009)
Supermarkets, hypermarkets	0.680 (0.099)	47.197	1.973 (1.625–2.396) ***
Age verification			
Yes	2.731 (0.078)	1213.520	15.345 (13.159–17.893) ***

OR: odds ratio. Reference: Lowest disposable income, Nonsummer vacation, Weekdays, Grocery stores, No age verification. * *p* < 0.05, ** *p* < 0.01, *** *p* < 0.001. Nagelkerke’s R squares = 0.431.

## Data Availability

Due to confidentiality agreements, supporting data can only be made available to bona fide researchers, subject to a non-disclosure agreement. Details of the data and how to request access are available from Health Promotion Administration, Ministry of Health and Welfare.

## Supplementary Materials

The following supporting information can be downloaded at: https://www.mdpi.com/article/10.3390/healthcare10102077/s1, Table S1: STROBE Statement—Checklist of items that should be included in reports of cross-sectional studies.
